# The molecular vista: current perspectives on molecules and life in the twentieth century

**DOI:** 10.1007/s40656-020-00364-5

**Published:** 2021-02-04

**Authors:** Mathias Grote, Lisa Onaga, Angela N. H. Creager, Soraya de Chadarevian, Daniel Liu, Gina Surita, Sarah E. Tracy

**Affiliations:** 1 Institut für Geschichtswissenschaften, Humboldt-Universität zu Berlin, Friedrichstraße 191-193, 10099 Berlin, Germany; 2 Max Planck Institute for the History of Science, Boltzmannstraße 22, 14195 Berlin, Germany; 3 Department of History, Princeton University, 129 Dickinson Hall, Princeton, NJ 08544 USA; 4 UCLA Department of History, 6265 Bunche Hall, Los Angeles, CA 90095-1473 USA; 5 ICI Berlin Institute for Cultural Inquiry, Christinenstraße 18/19, Haus 8, 10119 Berlin, Germany; 6 Department of History, Program in History of Science, Princeton University, 129 Dickinson Hall, Princeton, NJ 08544 USA; 7 Technoscience Research Unit, Faculty of Information, University of Toronto, 140 St. George Street, Toronto, ON M5S 3G6 Canada

**Keywords:** Molecular Biology, Historiography, Molecular Genetics, Structural Biology, Chemistry, Molecularization, Physical Science, Nutrition

## Abstract

This essay considers how scholarly approaches to the development of molecular biology have too often narrowed the historical aperture to genes, overlooking the ways in which other objects and processes contributed to the molecularization of life. From structural and dynamic studies of biomolecules to cellular membranes and organelles to metabolism and nutrition, new work by historians, philosophers, and STS scholars of the life sciences has revitalized older issues, such as the relationship of life to matter, or of physicochemical inquiries to biology. This scholarship points to a novel molecular vista that opens up a pluralist view of molecularizations in the twentieth century and considers their relevance to current science.

## Laboratory versus data? Historiography of life sciences beyond the gene

Michel Morange once remarked on the absurdity of trying to define molecular biology in abstract or transhistorical terms:

Molecular biology is not merely the description of biology in terms of molecules—if this were the case, it would not only include biochemistry, but also all those nineteenth-century studies in chemistry or in physiology, that led to the characterization of biological molecules. With such a broad definition, even Pasteur would have been a molecular biologist! (Morange 1998, p. 1, see also Morange 2020, p. 2).

Twenty-five years have passed since this reflection was first published. And while the inclusion of Louis Pasteur in the history of molecular biology still seems ludicrous, the boundaries of the field still—or once again—seem fluid. One reason for this may be that two decades after the completion of the Human Genome Project, a *longue durée* view of molecularization enables scholars to better situate the molecular gene as part of a broader endeavor to transform the material basis of biological research into large, portable data sets (Stevens 2013; Richardson and Stevens 2015; Leonelli 2016; Strasser 2019). The prominence of DNA sequencing and omics-approaches has increased the scale and pace of research through automation and bioinformatic analysis, and has produced a situation in which “doing life science” has become nearly synonymous with algorithmic thinking and computerized work (Roosth 2017; Liu 2017). Historiographically and historically, we have reached a point at which it is possible to think about this computationally-inflected history of genomics and proteomics as separate from, but related to, the history of molecular biology.

This historiographical review leads us up to the perceived data- and omics-centric present. It also emphasizes the points of historical friction and divergence from its dominance: behind the language of genetics, the dirty wet lab side of research has always provided a foundation. This admittedly presentist perspective sheds light on some historiographical absences that can prompt new research. Work on the technological frontier of the molecular life sciences includes contemporary philosophical reflections on tinkering with life in the age of synthetic biology and ethnographies of in silico or structural biology (Helmreich 1998; Myers 2015). Sophia Roosth (2017) has argued that there is a cohort of synthetic biologists who actively try to automate and suppress genetics at the wet bench. The question is whether it works (de Chadarevian 2018).

The genealogies of undervalued laboratory tinkering tap into biophysics, biochemistry, bioenergetics, colloid and surface chemistry, microscopy, and other endeavors that address the *terra incognita* in between cells and molecules. Even in “classical” molecular biology, including protein chemistry and structural studies (crystallography and electron microscopy) of proteins, viruses, or muscle fibers, chemistry was clearly a leading concern, although rarely trumpeted. In fact, John Kendrew, Max Perutz, Fred Sanger, Linus Pauling, and Gunther Stent were all chemists of some sort (Serafini 1989; de Chadarevian 2002; James 2007; Creager 2009). Relatedly, the range of physicists who participated in biology needs reevaluation. This task of reevaluation invites additional reflection upon how the narrative choices of historians might have reinforced the gendering of laboratory workspaces (Creager and Morgan 2008; Abir-Am 2014; Santesmases 2020), or restricted our purview to the west and the Global North in contrast to more transhemispheric understandings (Mignolo 2011; Mateos and Suárez 2014; Ling and Jiang 2019).

These aforementioned sensibilities, with which we now survey the history of the molecular life sciences, lead us to a vista that exhibits different sites of work and labor apart from university laboratories. It also includes diverse geographic regions, institutions, and actors that previously have been marginalized in historical narratives. In this essay, we re-examine the past in order to offer specific insights about areas that have been underattended to as part of the history of post-war molecular biology. In doing so, we opt to bypass the discourse of molecular biology’s disciplinarity by attending to perspectives that broaden the vista, since the longstanding preoccupation with the field’s origins is finally in the rearview mirror.^1^ Despite proclamations of a paradigm-shifting epigenetic revolution or of molecular biology's “evaporation” as a discipline (Rheinberger 2009), its “vapors”—the representations, imagery, metaphors, and scale of explanatory reasoning—are omnipresent in the life sciences, science education, and cultures of popular science.^2^

While the ancient question of how to relate ideas of “life” to those of “matter” persists, the rapid growth of molecular knowledge since the end of the nineteenth century has vastly outstripped the growth of integrative or synthetic conceptions of life (Liu 2019). Far from meta-scientific issues such as reductionism or the origin of life, the contributions summarized below highlight a set of questions about nutrition, energy, physiology, microstructure, and animal economy that predate or were concurrent with molecular biology. Below, we outline recent studies which, when examined together, suggest a continuation of research along these lines throughout the twentieth century, even during the perceived half-century of the hegemony of molecular genetics. Our own research questions that have arisen as a result of responding to the dominance of DNA narratives thus revise past narratives as much as they scope out an unexplored molecular vista. This essay resulted from a panel at the International Society for the History, Philosophy and Social Studies of Biology 2019 meeting in Oslo. Participants were Soraya de Chadarevian, Mathias Grote, Daniel Liu, Lisa Onaga, Gina Surita, and Sarah Tracy (presenting a paper co-authored with Hannah Landecker); Angela Creager commented.

## Structures of “Molecules” and “Life”

Historians have long examined how scientists have come to understand life on a molecular scale by focusing on their methods for visualizing and manipulating structural entities.^3^ In his paper, Daniel Liu addressed the question of “structure” in the longer history of molecularization reaching back into the nineteenth century, by analyzing efforts of a heterogenous group of scientists seeking to understand cells at submicroscopic scales. The apotheosis of this branch of molecular biology might be found in the use of electron microscopy to decipher the molecular structures of subcellular organelles, especially the mitochondria, Golgi apparatus, endoplasmic reticulum, and so on (see Rasmussen 1997a). This research continues as an auxiliary, rather than a central, part of cell biology and anatomy. The electron microscope was an instrumental extension of a research program that, prior to WWII, was often referred to as research on “fine structure” or even “sub-microscopic morphology,” using indirect imaging methods such as x-ray diffraction of whole cells in conjunction with polarized light microscopy and other inferential techniques in colloid chemistry. Rather than trying to understand atomic positions within a single molecule, as was the case in x-ray crystallography of protein and nucleic acid fibers, some of these researchers used x-ray diffraction diagrams of whole cells to show how layers of proteins and lipids were arranged, determine how thick each layer was, and obtain clues about the chemical identity of each substance composing these layers. As biophysicist Frank Schmitt put it, this technique could illuminate the “dimensions, configurations, and orientation of molecules” in cells (Schmitt 1944, p. 1587). This method was further combined with polarization microscopy, allowing analysis of birefringence and refractive index, revealing the “presence of oriented constituents in tissue systems, together with the direction of orientation, shape, crystallinity, partial volume and refractive index of the oriented components” (Schmitt 1944, p. 1587). X-ray diffraction was computationally intensive but yielded absolute dimensional and geometrical measurements, while polarization microscopy provided a more holistic picture of optical anisotropy, molecular orientation, and hints about material identity—and could be performed much more quickly and easily than x-ray techniques. The use of polarization microscopy to discern submicroscopic structures has a history stretching back to Carl Nägeli’s studies of starch granules in the 1860s, and was given new life once it was combined with early x-ray powder diffraction studies of cellulose in the 1920s.

Mathias Grote’s paper highlighted the continued impact of the combination of chemical and structural thinking in the colloidal “world of neglected dimensions” (Olby 1986, quoting Wolfgang Ostwald) in the molecular life sciences post-1970. Grote outlined a genealogy of molecular and colloidal practices in the chemiosmotic model of cellular energy generation that Peter Mitchell had proposed in the 1960s, which conceptually and practically linked interwar surface and membrane studies with late-twentieth-century bioenergetics. Grote showed how membrane-enclosed vesicles (liposomes) allowed biochemists from the early 1970s onward to *reconstitute* cellular structure in the test tube in order to spatialize biochemical reactions, such as the transfer of ions across a membrane. He argued that reconstitution—a concept and practice for the functional assembly of biological molecules into supramolecular structures—illustrates an interplay of modelling, understanding, and making components of life. Reconstitution, formerly employed in virus research (Creager 2002), and the resulting “plug-and-play” biology more generally, gained traction after 1980, and aimed at putting together and making work molecular components. Membranes, forming from lipids in aqueous solution by self-organization and by being re-formed, partitioned and inherited during the cellular life cycle, thus display exciting physico-chemical dynamics that have the potential to shake molecular biological certitudes. In the test tube, re-made membranes were fused with isolated proteins, analyzed by microscopy, studied functionally, and put together in different combinations, using synthesized RNA/DNA or recombinant proteins. Plug-and-play has also become fundamental to today’s synthetic biology, where the idea has been extended and commodified, e.g., in biobricks or ongoing projects to create synthetic cells (Grote 2019). Moreover, plug-and-play has brought physiology and molecular biology (of physical, chemical, and genetic sorts) into close contact, and has in fact rendered them indistinguishable in many fields, bridging gaps between the molecular, supramolecular/colloidal, and cellular levels of structure. The bacterial cell wall, both as an ultramicroscopic structure and an object of metabolic research in the context of penicillin action, is a related, earlier example for such border crossings. Its research has juxtaposed fields addressing different levels of biological organization and helped create a “chemistry of shape” (Santesmases 2016, p. 29).

Even the history of genetics, and not just that of DNA, is not immune to the historical importance of structural methods and entities. Soraya de Chadarevian showed that starting in the late 1950s cytogeneticists were able to correlate hereditary diseases with alterations of chromosome structure that were visible under the light microscope, offering important diagnostic tools to medical geneticists. Even though molecular biologists predicted for decades that sequence data would displace older techniques relying on observations of chromosomes under microscopes, that day never arrived. Rather, cytogeneticists proved able to visualize complex mutational events that are hard to identify using the tools of molecular biology. As it turns out, many of these complex chromosome-level mutations (such as translocations and inversions) are key genetic signatures of cancer cells, making cytogenetics especially valuable in oncology and cancer research. Her work suggests that scientific fields do not simply operate as political regimes that replace each other; more often, specialties continue on parallel tracks, and the public visibility of one discipline need not spell the demise of others (de Chadarevian 2020). The dynamic three-dimensional structure of chromatin fibers is gaining new salience in studies of epigenetic regulations in the cell, indicating that even in the field of molecular genetics there is much to gain from a perspective that includes structural and cellular next to environmental considerations (Landecker 2015).

In addition, methods of structure determination display a greater heterogeneity and topics such as the relation of molecular and cellular structure a greater continuity than previously thought. Beyond x-ray crystallography of protein or DNA, membranes, chromosomes, and other subcellular structures were analyzed by a variety of such methods. Furthermore, new methods, such as fluorescence microscopy, optical and magnetic spectroscopies, or cryo-electron microscopy were developed; the latter has been in the limelight since the 2017 Nobel prize to Jacques Dubochet, Joachim Frank, and Richard Henderson (Grote 2019; Reinhardt 2017).

## Lively economies, metabolism, and interdisciplinarity

While this focus on structure has provided scholars with a useful vantage point, there are other avenues into the lesser-visited corners of the history of twentieth-century biology: these include studies of the prolific use of metaphors in the life sciences (besides the gene-as-code-metaphor); the history of lower-status fields, such as, nutrition science; and analyses of biological phenomena such as symbiotic relationships. The study of metaphorical language illuminates how historical actors understood and communicated conceptualizations of life as well as its experimentally known underpinnings (Keller 1995). The most commented-upon metaphor in molecular biology has been that of genetic material as “code” or “information,” perhaps demonstrated most comprehensively by Lily Kay’s *Who Wrote the Book of Life?* (2000).^4^ But as Andrew Reynolds (2018) has shown, cell biologists drew on a range of other metaphors. Since the nineteenth century, cells have been cast variously as “organisms,” “citizens,” “machines,” and “factories” (Reynolds 2018; Nyhart and Vienne 2017). Gina Surita’s paper examined the metaphors used by biochemists to understand subcellular life. For example, the history of bioenergetics can be understood as the gradual articulation of the cell as a kind of “economy,” in which the universal energy “currency” of ATP (adenosine triphosphate) circulated in order to “pay” for various life-sustaining, energy-requiring metabolic reactions (Gina Surita, Ph.D. dissertation in progress). Drawing upon older, physiological invocations of the metaphor of the “animal economy,” twentieth-century bioenergeticists localized this notion of a vital economy to the cell, where, incidentally, the vast majority of energy exchanges were thought to take place in the cell cytoplasm, outside the nucleus.

Neglected approaches to “molecules” and “life” also come into view when historians survey low-status areas of research. Nutrition is especially important in this regard—the association of life with chemical transformation and metabolism runs from nineteenth-century animal economy right through the industrialization of food production in the twentieth century (Kamminga and Cunningham 1995; Stoff 2012). Following the researchers who determined basic nutritional requirements for humans, animals, and plants reveals a bundle of practical ties between molecular analysis and agriculture (Kollmer 2020). As Lisa Onaga (2021) shows, the biochemical study of the nutritional requirements of silkworms in Japan grew in the 1930s as an outcome of falling silk prices in the country. Scientists in Japan began to investigate how to supplement limited supplies of mulberry leaves with nutrient extracts of mulberry and soybeans to feed silkworms. At the same time, many farmers converted mulberry acreage to other crops and many others emigrated to the puppet-state of Manchuria to work on soybean plantations. The manufacture of molecularly-formulated artificial silkworm feed involved chemical studies of nutritional factors responsible for physiological feeding behaviors of silkworms, and contributed to the broader history of making artificial media for cultivating laboratory organisms.

Historians of biology have also begun analyzing how theories of metabolism fed into the development of chemically-defined media and animal feed (Landecker 2016a, 2019). These histories connect to the vibrant literature on model organisms, with its focus on how animal and plant systems serve as laboratory exemplars for understanding life, and they also reflect more recent attention to scientific infrastructures as well as issues of animal welfare.^5^ For example, scholars involved in the Animal Research Nexus, a 5-year, Wellcome Trust-funded collaborative project in the UK, have been undertaking an interdisciplinary and reflexive examination of how laboratory biomedical researchers implement animal models while weighing matters of protecting and promoting both human health and animal welfare (Friese 2018; Davies et al. 2020). These inquiries dovetail with what could be called the molecularlization of agriculture and animal husbandry. Be they mice or sheep, twentieth-century investigations of molecular life processes in agriculture and biomedical settings relied on certain configurations of infrastructure, finance, and labor (García-Sancho and Myelnikov 2019).

One of the limitations of earlier studies of molecular biology has had to do with how sociocultural, political, and economic dimensions were brought into the narratives as a consequence of how those studies were attuned primarily to molecular biological narratives. Dominic Berry’s work on synthetic biology illustrates a newer approach to integrating the history of molecular biology with histories of technology and business. By using material and discursive analysis to study how objects, institutions, machines, journals, companies, human actors, and molecules related to other, he ascertains the significance of multiple meanings of “making” DNA. His strategy to locate a lesser-known, engineering-centered narrative serves to avoid uncritically reproducing the sanctity of DNA (Berry 2019). Such cognizance also works against the commonplace narrative that the commercialization of biology mainly concerns biotechnology since the 1980s. Historical research emerging from collaborative projects such as “Organisms and Us” at the University of Adelaide highlights how organisms of longstanding commercial relevance have been gaining scientific attention while the molecular pathways of organisms adapted to extreme environmental conditions have offered strategic biological appeal to scientists (Dietrich et al. 2020; Green et al. 2018).^6^

The food and agriculture aspects of molecular biology have also given rise to discussions about molecularization as a strategy *within* Science & Technology Studies (STS) that advocates for greater attention to key molecules as regulators. Aligned with these calls for diversification of which objects count as historically relevant agents, Sarah E. Tracy has examined research that aimed to measure the effects of the flavor chemical monosodium glutamate (MSG). While glutamates are naturally occurring compounds, they are also common food additives, and their effects have been a source of controversy since the late-1960s. Tracy’s paper pointed to the striking compartmentalization of research on MSG. On the one hand, diabetes researchers have relied upon the obesogenic effect of large doses of MSG upon newborn mice. On the other hand, food scientists have pursued the potentially advantageous appetitive and digestive effects of MSG, based on findings from adult rodent models (Tracy and Landecker, forthcoming). Key connections between the flavor industry’s objectives and the risk of metabolic disorder due to food additives have been overlooked as a result of the compartmentalization of research and the complexity (e.g., the developmental and species variability) of glutamate’s bioactivity (Tracy 2018, 2019). As Tracy remarked, recent attention in biomedicine to metabolic disorder illustrates that genes are not the only informational actors in the body (Landecker 2011, 2016b).^7^

The effects of molecules like glutamate in rodent studies of digestion and metabolism point us to emergent historical and philosophical debates on how to study so-called “postgenomic” biology. The epigenetic processes that inform holobiontic relationships in organisms have generated much attention that have called into question not just the stability of DNA but the notion of the organism itself (Dupré and O’Malley 2013; Baedke et al. 2020).^8^ Feminist scholars of bioscience, for instance, have suggested including the metabolic contributions of bacteria to the biological processes of other organisms, including humans, in order to recognize the historical roles of bacteria in scientific knowledge production (Roy 2018). Furthermore, studies of social epigenetics linked to diet and analyzed relative to disease susceptibility have injected scholarship with new evidence that affords the articulation of relational ideas of race, environment, and society (Baedke and Nieves Delgado 2019). Molecular-scale understandings of metabolism also help broaden our historical perspective into the historical sciences. Disciplines like zooarchaeology are increasingly building context- and actor-dense reconstructions of the evolution of humans, the animals they have depended upon (for nourishment, clothing, tools, and labor), and the microbiota hosted by them both. New fields like bioarchaeogenetics have sought to understand the relationships among animals, plants, and microorganisms from micro- to macro-scales by integrating genomic and proteomic diagnostic tools into existing methods in biology and ethnography (Sykes 2014; Hendy et al. 2018).^9^ Historians of the life sciences face a special responsibility to interrogate how such technologies are used to articulate longue durée bioarchaeological narratives, even as we are called upon to contribute to this new and exciting area of scholarship.

## Rethinking biology and the physical sciences

In various ways, the recent scholarship we highlight illustrates the importance of the physical sciences, and especially chemistry, to biology. By contrast, conventional accounts attribute the origins of molecular biology to physicists who turned their attention to solving the secret of life, especially after the devastating use of atomic bombs at the end of World War II. Attesting the critical role of physicists was always partisan, and even though Francis Crick and Maurice Wilkins certainly read and were inspired by Schrödinger’s *What is Life?* (Rasmussen 1997b), they came away with different conceptions of how biology and physics ought to engage each with other. Although this scholarship illustrates the enduring importance of the physical sciences to biology, we also want to highlight that we are defining “the physical sciences” much more broadly than most older histories of molecular biology did: we understand the physical sciences to include the chemical and material sciences as well. In this we are following a similar shift happening in other areas of the history of physics more broadly: For example, Schwartz (2004, 2008) has shown how the central role of chemists in Manhattan Project was marginalized by a combination of the project's secrecy and post-Hiroshima public relations, while Joe Martin (2018) has shown how solid state physicists and materials scientists navigated the shifting divide between physics and chemistry after World War II. By contrast, the narrative of physicists revolutionizing biology after reading Schrödinger and in the aftermath of Hiroshima was a mythology that drew a bright line between a valorized “pure” atomic or quantum physics and an impure “applied” or industrial physics and chemistry (Delbrück 2007; Reinhardt 2018). In fact, the influx of physical scientists into biology in the twentieth century was always as much from chemistry as physics, and it has long been noted that the adoption of tools and approaches from the physical sciences by biologists themselves also heavily tilted towards chemistry (Kohler 1976; Abir-Am 1982; Keller 1990; Kay 1993; Deichmann 2007).^10^ More than two decades ago, Rasmussen (1997b) persuasively argued that biologists pulled physics in as much as physicists pushed into biology; in some cases, the physics involved had turned out to be so esoteric and strange that historians and philosophers have struggled to make sense of it (Sloan and Fogel 2011). Paying attention to practice rather than rhetoric in biology brings into view chemical tools, techniques, and approaches, as well as those from physics that are not prominent in the historiography, such as the solid-state physics, surface science, and microstructural studies (Martin 2018).

Biologists often turn to chemistry for very pragmatic reasons. As Angela Creager (2017) observed in an essay on her “chemical reaction” to this historiography, when biologists handle, purify, stabilize, and analyze the stuff of living organisms they generally find themselves doing chemistry—even when they don’t remark on this in their publications. This insight seems especially apt for the other quite variegated cases that composed our panel. Not all of these papers were about biochemistry, but even colloidal chemistry made an appearance in the session, and twentieth-century nutritional studies relied heavily on analytical chemistry (on organic chemistry in hormone, vitamin, and enzyme studies, see e.g. Stoff 2012; Schürch 2017).

Earlier historical works in the field included a great deal of chemistry but did not always emphasize it. Take, for instance, the profound historical studies of virus research, structural biology of DNA and proteins at Cambridge, or protein synthesis published around the millennial heyday of the Human Genome Project (Creager 2002; de Chadarevian 2002; Rheinberger 1997), which show how thinking in terms of code and information has always been intertwined with material, chemical analyses, especially when it comes to hands-on laboratory practice. The attention to chemical thinking and working is even more prominent in literature from before this period, such as work on physiology and biochemistry by Holmes (1974) or Robert Kohler (1982). Together with new interest in materials and metabolism (Landecker 2011, 2019), chemistry has become an unintended beneficiary of recent historiographical developments. This aligns with Creager (2017), who observes that focusing on materials-centered research challenges a strong divide between biology and chemistry.^11^ In this sense, the recurrent importance of structures in the papers by our panelists reflects more than acknowledging the essential chemical toolbox. That said, the focus on structures also closes the loop with respect to the historiography of molecular biology and physics that we mentioned at the outset, by making the chemistry in the “structural school” of molecular biology more visible (Kendrew 1967).

In the historiography of molecular structural research there is much to be done, if only to gain a firmer grasp of the sheer extent of molecularization: It was not only DNA and globular proteins whose structures were resolved down to the atomic level to such great fanfare. For example, as Karl Matlin (2016) has recently shown, theories of the structure of mitochondria were essential in deciphering the spatially complex synthesis of ATP. Similar studies have yet to be done on the history of transport theories, chloroplasts and related plant plastids, the cytoskeleton, etc. Far from merely cataloging the histories of different structures, such studies can illustrate the diverse ways in which theories of structure and theories of physiological or biochemical function have been forged. After all, *every* living thing, every *part* of every living thing, and every *derivative* of every living process can be said to be ultimately made of molecules! To what extent this brings us full circle back to the problem of a definition of molecular biology highlighted by Morange is a question we do not want to resolve here. Our point is that we believe our vista of the molecular life sciences must be broadened to accurately capture the history of twentieth century biology.

## Conclusion

As some of our contributors made clear, rethinking molecular biology’s history is about more than the borders with the physical sciences or the importance of heredity. Much new work is looking closely at research previously deemed “old-fashioned” or marginal—in part because the now seemingly central problems of (largely genetic) data, computing, or even machine learning in biology have achieved a significant degree of abstraction and independence from the material problems that historically occupied molecular biologists. As Soraya de Chadarevian asked off-handedly during the session, “Is this because genomics is no longer a chemistry-adjacent science?” Additional questions raised by the audience highlighted interests and concerns about the intersections between molecular biology and industry. We believe these types of questions and discussions are neither features limited to our panel nor circumstantial, but indicate broader shifts afoot.^12^ Personalized medicine, nutrition, genetics, animal science, even cosmetics, are back in view (e.g. Boniolo and Nathan 2017). The efforts in several quarters to better understand feeding, whether of silkworms, cells, or people, are especially striking (Landecker 2016a).

The new attention to lower-status fields can also help make visible scientists of different socioeconomic *or* educational backgrounds, and from underrepresented groups, genders, and ethnicities in the doing of science, including molecular biology. The foregrounding of marginalized actors, be they women, persons of color, or scientists from the Global South, can help diversify the curricula that is used to teach about the history and philosophy of the life sciences to new generations and thus impact whose histories are included and how those histories are remembered (Spanier 1995; Wailoo 2001; Wailoo and Pemberton 2006; Zulueta 2009; Hartley and Tansey 2015; Onaga 2014; Jiang and Stevens 2015; Nelson 2016a).^13^ Attention to the subordination that is associated with scut work may help us understand the gendered dimension of categorizing “ideal” scientists as those who work with their minds, not their bodies. Molecular equivalents of this gendered division of labor abound; as Hannah Landecker (2013, p. 501) has observed, the “housekeeping” functions of the cell, often used to describe the various processes associated with cellular metabolism, have long been demarcated from and seen as subsidiary to the “executive” genetic functions of the cell.^14^ Overcoming these norms takes deliberate attention. Much as historians of technology have highlighted women in computing (e.g., Hicks 2018), it is necessary to recognize the names and faces of those women who have worked in molecular biology, from Margaret O. Dayhoff and her work on computational sequencing (Strasser 2010) to technicians and postdoctoral fellows (e.g., Martha Chase and Susan Berget) who contributed to path-breaking laboratory experiments. Detailed recognition of these individuals alongside women scientists like Esther Lederberg, June Almeida, Marie Maynard Daly, Mildred Cohn, Louise Chow, Susan Lindquist, Tu Youyou, Carol Greider, and Elizabeth Blackburn, to name a few, can thus draw out the harder realities of where the molecular labor resides in biology. This, in turn, can allow us to map the intellectual power dynamics among many more players in just as many neglected corners of science.

Since the development of high-throughput genome sequencing in 2006, scientists have been increasingly doing cutting-edge biology in silico, relegating the bench to background labor (Stevens 2013; Roosth 2017). Looking back, somewhere in between the characterization of DNA and in silico biology, there was a great deal of laborious, dirty—and at times dangerous—wet lab research that is easily forgotten next to burnished double helices and computers. Equally invisible are the geographies and labor involved in procuring biological material used in research, for example, the viscera of pork from industrial farms, or the re-using of kitchen waste (Blanchette 2020; Ibáñez Martín and de Laet 2018). These issues of life and labor are the other side of the coin to philosophical questions that have arisen about how biology as data, models, and changing norms of experimentation give rise to personalized medicine and the associated problems that can ensue (Ratti 2020; Green et al. 2019). The material work, due to its often idiosyncratic and sometimes personal character linked to specific availabilities, skills, equipment or traditions, also helps to further trace the “power of place,” such as by research carried out in very specific labs or beyond them, in companies, hospitals, or the field, and take into account the impact of the economic and political conditions of the molecular life sciences (Fischer 2013; Curry 2014; Santesmases and Suárez Diáz 2015). In addition to the fact that the landscape of the history of molecular biology is more diverse and messier than previously imagined, some fellow scholars are venturing beyond its marked paths altogether. To be sure, much of the terrain remains unmapped, but this suite of papers provided a few snapshots of the vistas for other scholars interested in how scientists sought to relate molecules and life in the twentieth century.

## Footnotes

1 Excellent work on this point exists already (e.g., Abir-Am 1992; de Chadarevian and Gaudillière 1996; de Chadarevian and Kamminga 1998); de Chadarevian and Rheinberger 2009).

2 See Morange (2020, pp 1–8; 13–22; 369–386).

3 For example, tools from the physical sciences used to develop the Tiselius electrophoresis apparatus not only separated proteins within a mixture, but enabled their photographic visualization. See Kay (1988).

4 For a different perspective on the “Book of Life Metaphor,” see Brandt (2005).

5 For key examples of recent and established model organisms literature, see Kohler (1994), Rader (2004), Endersby (2007), Ankeny (2010), Leonelli and Ankeny (2013), Ankeny and Leonelli (2019). On animal welfare, see Nelson (2016b).

6 The project investigators are Rachel Ankeny, Sabina Leonelli, and Michael Dietrich. See https://arts.adelaide.edu.au/organisms-and-us/.

7 For related discussion, see Oudshoorn (1990), Burian (1996).

8 The German Research Foundation (DFG)-funded research group Return of the Organism (PI: Jan Baedke) at Ruhr University Bochum investigates an array of questions surrounding organisms known at their genomic levels by bringing philosophical, historical, and social and anthropological methods into conversation. https://rotorub.wordpress.com/.

9 The Proteins and Fibers working group at the MPIWG, including our co-authors Lisa Onaga, Daniel Liu, and Soraya de Chadarevian, has been examining the historical implementation of molecular biology and chemistry in interdisciplinary scientific fields that assay samples derived from animal materials and body parts (e.g., teeth, bones, hides). Historical reconstruction of the science contributing to “textbook” cases of gene-culture co-evolution such as lactase persistence is, for example, facilitated by analyzing how changing suites of methods and technique came to include microbial information. See https://www.mpiwg-berlin.mpg.de/research/projects/proteins-and-fibers-scaffolding-history-molecular-signatures.

10 The role of the Rockefeller Foundation, and especially Warren Weaver, in encouraging interdisciplinary research that brought the between the physical and life sciences—which really meant involved bringing the former into the latter—has been well-documented and critiqued since Robert Kohler’s classic 1976 article on Weaver.

11 Reinhardt (2018) makes this point more broadly for how a materials-based approach to history shows that much of modern science is saturated with chemistry, even when it is not explicitly connected with that discipline.

12 The 2019 formation of the Biological Engineering Collaboratory, spearheaded by Dominic Berry, Janella Baxter, and Robert Smith, for instance, has established a network of scholars guided by a mission to encourage integrative studies of biology and technology: https://www.bioengcoll.org/.

13 This concern is revisited in “Statement on Racialized Violence and Resources,” *HSS Graduate and Early Career Caucus* (blog), July 13, 2020, https://hssgecc.wordpress.com/statement-on-racialized-violence/. It lays out “a vision of a diverse profession, which includes not only promoting the voices and scholarship of more Black historians of science, but also calling on white and non-black historians of science to recognize their own privilege.” Within the history of the molecular life sciences, recognizing that dominant historical narrative accounts of DNA have routinely elided scientists of color (be they students, technicians, or full professors) will be a key strategy toward the articulation of underknown histories.

14 This contrasts nicely with Evelyn Fox Keller’s analysis of “master molecule” narratives in descriptions of gene action, in which executive metaphors are prominent (Keller 1995).
